# Calcium Homeostasis and ER Stress in Control of Autophagy in Cancer Cells

**DOI:** 10.1155/2015/352794

**Published:** 2015-03-03

**Authors:** Elżbieta Kania, Beata Pająk, Arkadiusz Orzechowski

**Affiliations:** ^1^Electron Microscopy Platform, Mossakowski Medical Research Centre, Polish Academy of Sciences, A. Pawińskiego 5 Street, 02-106 Warsaw, Poland; ^2^Department of Physiological Sciences, Faculty of Veterinary Medicine, Warsaw University of Life Sciences (SGGW), Nowoursynowska 159 Street, 02-776 Warsaw, Poland

## Abstract

Autophagy is a basic catabolic process, serving as an internal engine during responses to various cellular stresses. As regards cancer, autophagy may play a tumor suppressive role by preserving cellular integrity during tumor development and by possible contribution to cell death. However, autophagy may also exert oncogenic effects by promoting tumor cell survival and preventing cell death, for example, upon anticancer treatment. The major factors influencing autophagy are Ca^2+^ homeostasis perturbation and starvation. Several Ca^2+^ channels like voltage-gated T- and L-type channels, IP3 receptors, or CRAC are involved in autophagy regulation. Glucose transporters, mainly from GLUT family, which are often upregulated in cancer, are also prominent targets for autophagy induction. Signals from both Ca^2+^ perturbations and glucose transport blockage might be integrated at UPR and ER stress activation. Molecular pathways such as IRE 1-JNK-Bcl-2, PERK-eIF2*α*-ATF4, or ATF6-XBP 1-ATG are related to autophagy induced through ER stress. Moreover ER molecular chaperones such as GRP78/BiP and transcription factors like CHOP participate in regulation of ER stress-mediated autophagy. Autophagy modulation might be promising in anticancer therapies; however, it is a context-dependent matter whether inhibition or activation of autophagy leads to tumor cell death.

## 1. The Outline of Autophagy

Autophagy pathway is unique and is characterized by the appearance of double- or multiple-membrane cytoplasmic vesicles which absorb the bulk of cytoplasm and/or cytoplasmic organelles such as mitochondria and endoplasmic reticulum to be destroyed by the lysosomal system of the same cell [1, 2]. Autophagy begins with enwrapping the cytoplasmic constituents by membrane, which originates from ER, Golgi apparatus or is formed de novo through nucleation, assembly, and elongation of small membrane structures. Closure of these membranes results in the formation of double-membrane structure called autophagosome. In the next step autophagosome fuses with late endosome (multivesicular body) or directly with lysosome and generates amphisome or autolysosome, respectively. Subsequently, amphisome as transient form also fuses with lysosome. Finally, the lysosomal hydrolases degrade the cytoplasm-derived contents inside the autolysosome together with its inner membrane [3]. Autophagosomal membrane formation requires a multiprotein complex that consists of Beclin 1 (Atg6), class III PI3K (Vps34), and p150 myristoylated protein kinase [1] as well as Vps15, UVRAG, Bif1, and Ambra 1 [4]. Another complex involved in autophagosome formation is focal adhesion kinase (FAK) family interacting protein of 200 kDa, Unc-51-like kinase 1/2, autophagy-related gene 1/13 (FIP200-ULK1/2/Atg1/13) [5]. Further elongation is mediated by two ubiquitin-like conjugation systems based on conversion of microtubule-associated protein 1 light chain 3 (MAP LC3) from the free form (LC3 I) to the lipid-conjugated membrane-bound form (LC3 II) [6]. Although autophagy is thought to be mainly nonselective degradation mechanism, latest studies report the presence of specific receptors and other selective adaptor proteins sensitive to organelle injury or aggregated proteins [3, 7].

## 2. The Role of Autophagy in Cancer Cells

Autophagy is the major cellular route for degrading long-lived proteins and cytoplasmic organelles to provide the energy required for minimal cell functioning when nutrients are scarce or not available [1, 2]. The catabolic advantage of increased autophagy may be also critical in various stress conditions, for example, hypoxia, growth factor deprivation, starvation, ER stress, ROS accumulation, protein aggregation, and numerous anticancer treatments. Therefore, autophagy in mammalian cells serves as an adaptive mechanism and is activated when cell is prone to die, to recycle amino acids and macromolecules necessary for cell survival. The role of autophagy in cancer cells, however, is still under investigation. It seems that autophagy function depends on several factors, for example, step of tumor formation, tissue origin, and gene mutations existing in specific cancer type. Some cancer types like human pancreatic cancers with constitutive Ras activation have elevated levels of autophagy that contributes to their growth and survival [8]. Conversely, other tumor types like human breast, ovarian, and prostate cancers have allelic deletions of the essential autophagy regulator Beclin 1, indicating that decreased autophagy may promote tumor development [9]. Overall the significance of autophagy in tumors can be distinguished into two functions. Firstly, autophagy can play a tumor suppressor role by maintaining cellular fidelity and, if necessary, contributing to cell death execution. Secondly, autophagy may exert oncogenic effects by promoting tumor cell survival and preventing cell death. Both roles of autophagy in tumor development will be discussed in this review.

Autophagy can play protective roles in early stages of cancer development by eliminating aggregated proteins or damaged organelles, preserving cells from further damages [2, 10]. Moreover oncosuppressive function of autophagy is manifested by limiting chromosomal instability, reducing oxidative stress, preventing intratumoral necrosis and local inflammation, and supplying nucleotides for DNA replication and repair [10, 11]. Several proteins involved in autophagy regulation are actually described as oncosuppressors. For instance, the UVRAG protein, monoallelically deleted at high frequency in human colon cancers, interacts with Beclin 1 to form a class III PI3-K signaling complex, the initial step in autophagosome formation [12]. Another function of autophagy in preventing of tumor progression is its ability to regulate cell death processes. It is known that autophagy and apoptosis share some regulatory pathways including proteins such as Bcl-2, Bcl-X_L_, cFLIP, caspase 3, tBid, Bad, and PUMA [8, 13]. In many cases execution of apoptosis even depends on autophagy. During the DNA damage, expression of macroautophagy regulator DRAM-1 is required for p53 mediated apoptosis [14]. In NIH 3T3 spontaneously immortalized cells inhibition of B-Raf by UI-152 was specifically cytotoxic to v-Ha-Ras-transformed cells and evoked both autophagy and apoptosis. Inhibition of autophagy by 3-MA did not rescue transformed cells from cell death indicating the cooperation between autophagy and apoptosis pathways. Another example is autophagy and apoptosis induction upon carnosol treatment both* in vitro* and* in vivo* in triple-negative breast cancer (TNBC) [15]. Surprisingly, autophagy in these studies was Beclin 1-independent, which, according to the authors, might be responsible for death-stimulating effect of autophagy. Autophagy might also participate in necrotic type of cell death [13]. In pancreatic cancer cells PANC-1, a derivative of allocolchicine, Green 1 [(S)-3,8,9,10-tetramethoxyallocolchicine], caused necrotic cell death that was autophagy-dependent [16]. These processes occurred selectively in cancer cells and autophagy was induced in response to elevated ROS levels after Green 1 administration. Furthermore, many authors even refer to autophagic cell death or type II programmed cell death. In apoptosis deficient tumor cells, autophagy is induced to maintain cell metabolism and viability during nutrient starvation and protect cells from necrosis. Ultimately, if the nutrient deprivation persists, prolonged autophagy may lead to autophagic cell death [17]. Xiong et al. [18] reported 5-FU (5-fluorouracil) induced autophagic cell death in Bax and PUMA deficient HCT116 colon cancer cells which were apoptosis defective. Furthermore, autophagy inhibition by 3-MA resulted in decreased cell death rate [18]. In hepatocellular carcinoma cell lines HepG2 and HuH-7 and line xenografts treated with cannabinoids (Δ9-tetrahydrocannabinol, JWH-015) autophagy was mediated by CaMKK*β*-AMPK and led to apoptosis. In these studies, blockage of autophagy also impeded apoptosis [19]. These results suggest autophagy to be supporting or alternative to apoptotic cell death pathway.

In established tumors, autophagy may conversely exert an oncogenic effect by preventing tumor cell death. Autophagy can limit the cytotoxicity of tumor necrosis factor superfamily (TRAIL), can suppress p53 response induced by DNA damage, and can sustain mitochondrial metabolism and stress tolerance [10, 20]. Thus, inhibition of autophagy often sensitizes cancer cells to apoptotic, necrotic, or necroptotic cell death [8, 21]. It was shown that in the presence of a potent chemotherapeutic agent, cisplatin, esophageal cancer cell line EC9706 induced autophagy through class III PI3-kinase pathway. Although cell growth was effectively inhibited in time- and dose-dependent manner, additional treatment with autophagy inhibitor 3-MA potentiated cell growth inhibition and induced apoptosis [22]. Under stress conditions, DNA repairing enzyme, PARP-1, massively synthesizes poly-ADP-ribose and this causes the decrease in cellular NAD^+^ and ATP levels [23]. Insufficient ATP production to maintain plasma membrane integrity may induce metabolic catastrophe and cell lysis [17]. A rapid drop in ATP is also a feature of proceeding necrosis that can be abolished by providing necessary sources for ATP synthesis by autophagy. However, tumor cells have commonly inactive mechanisms of apoptosis induction and constitutively active PI3-K pathway, being responsible for cell growth and proliferation. These types of tumors cannot induce autophagy (active mTOR) in response to metabolic stress, which may lead to necrotic cell death [17].

Taken together, autophagy is currently considered as a possibly important mechanism to be used in anticancer therapy. However, possible role of autophagy in both oncogenesis as a survival promoting factor and tumor prevention as a death inducing factor should be seriously considered.

## 3. Molecular Pathways Related to Autophagy Induction in Cancer Cells

There are several known pathways leading to autophagy activation in cancer cells. Beclin 1 has been first identified as a Bcl-2 interacting protein [12]. Originally the contribution of the Bcl-2 family in tumorigenesis was limited to modulate apoptosis but recently there are also evidences to their function in control of metabolic processes including autophagy. In fact, the role of antiapoptotic factors like Bcl-2, Bcl-X_L_, and Bcl-w is to suppress autophagy, mainly by interacting with Beclin 1 [12]. Accordingly, the inhibition of Bcl-2 was shown to induce autophagy in multiple tumors [12, 24]. Furthermore, proapoptotic BH3-only proteins such as Bad, Bik, and BNIP3L are also described as autophagy inducers, acting by releasing Beclin 1 from the inhibitory action of Bcl-2 protein [12]. Bcl-2 is mainly located on mitochondrial, nuclear, and ER membranes and can affect autophagy in at least two ways: by directly binding to Beclin 1 or possibly by binding to IP3-R and regulating the Ca^2+^ level in ER [25].

**Figure 1:**
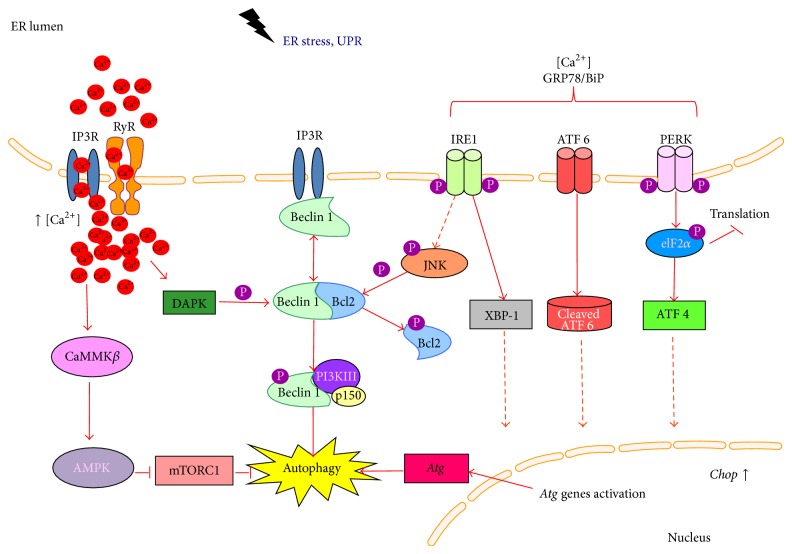
Autophagy mediated via ER stress and UPR activation. The figure represents proposed scheme for autophagy modulation in cancer cells through ER stress and UPR activation.

The main molecular mechanism of autophagy occurs via repressed activity of mTORC1, which consists of serine/threonine protein kinase, mammalian target of rapamycin (mTOR), its regulatory associated partner, Raptor, PRAS40, and mLST8 [26]. Direct target of mTORC1 inhibition is ULK complex; however, its phosphorylation might be regulated also independently of mTORC1 activity [3]. The mTORC1 activity is regulated by distinct molecular pathways. It might be activated via class I PI3-K-Akt pathway which is sensitive to growth factors and transduces signal to activate cell growth and proliferation [17, 27] or by Raf-1-MEK1/2-ERK1/2 pathway, which contribute to amino acid depletion-induced autophagy [2, 28]. Nutrient deprivation and calcium homeostasis disturbances can influence mTORC1 activity as well. AMP-activated protein kinase (AMPK) is a mediator of autophagy in such circumstances. It might be activated either by increased AMP/ATP ratio inside the cell during starvation or possibly by CaMKK*β*-dependent pathway in response to elevated calcium levels in cytoplasm [6]. Finally, when mTORC1 complex is inhibited, it relocates from lysosomes that proceeds autophagy and allows nuclear translocation of dephosphorylated transcription factor EB (TFEB), which in turn activates the* Atg* genes [3]. Both starvation and calcium perturbations may lead to activation of UPR cascade and ER stress. This review focuses mostly on involvement of calcium homeostasis and glucose deprivation in ER stress-mediated autophagy induction in cancer cells (Figure 1).

## 4. Involvement of Ca^2+^ Homeostasis in Autophagy Induction

Calcium homeostasis is regulated by several calcium channels. Plasma membrane calcium ATPases (PMCA) are located in plasma membrane and actively pump Ca^2+^ outside the cell. Ca^2+^ release-activated Ca^2+^ channels (CRACs) are also located in plasma membrane and mediate the store-operated calcium channel entry (SOCE) [29]. CRACs are activated by Ca^2+^ released from ER by channels formed by IP3-R and RyR receptors. Ca^2+^ redundancy in cytoplasm is actively transported backward into ER by sarco-/endoplasmic reticular calcium ATPase (SERCA), which is a membrane pump located in ER. Several voltage-dependent calcium channels are also reported to participate in calcium homeostasis regulation. Specific voltage values are required to activate dedicated types of calcium channels: L, N, P, Q, R, and T.

Ca^2+^ is one of the most important regulators of cell survival/death processes. As a second messenger, Ca^2+^ is able to activate or inactivate various regulatory proteins such as enzymes, transcriptional factors, or molecular chaperones. It was shown previously by several authors that the disorder of calcium homeostasis can evoke different types of cell death in cancer cells. There are evidences that *β*-lapachone induces *μ*-calpain-mediated and is independent of caspase activity cell death in MCF-7 cells [30]. Pajak and Orzechowski [31] reported the proapoptotic effect of calcium ion chelators EGTA and EDTA in COLO 205 adenocarcinoma cells. Høyer-Hansen and Jäättelä [25] observed autophagic cell death induced by factors increasing in different manners the cytosolic Ca^2+^ such as vitamin D3 and its chemotherapeutic analog EB 1089, ATP, ionomycin, and thapsigargin in MCF-7 breast cancer cells. In Ca^2+^-dependent induction of autophagy, Ca^2+^ released from intracellular stores or fluxed from extracellular space via distinct calcium channels activates CaMKK*β*, which mediates AMPK-dependent inhibition of mTORC1 [6].

One of the best investigated mechanisms of autophagy induction, involving modulation of calcium channels activity, is IP3-R-Beclin 1-Bcl-2 pathway [4]. This pathway is mTOR-dependent and mediated through ER stress and UPR activation. IP3-R, the receptor for major cellular second messenger IP3, is known for regulating apoptosis signaling, although its inhibition is also described as an event leading to macroautophagy induction [4]. Beclin 1 promotes autophagosome formation by interacting with class III PI3-K, p150 myristoylated kinase, and other proteins. Creation of autophagy promoting complex can be abolished by competitive Beclin 1 interaction with IP3-R or Bcl-2. In fact, the application of xestospongin B, the antagonist of IP3-R, caused autophagy due to disruption of IP3-R and Beclin 1 complex. Moreover, the activity of this complex might be increased or inhibited by overexpression or knockdown of Bcl-2 which can be ectopically expressed in ER and interact with IP3-R-Beclin 1 [4]. Thus, when IP3-R serves as a scaffold protein, lowering its level may trigger autophagy [4, 32]. Inhibition of inositol monophosphatase by lithium chloride (LiCl) also evoked IP3-R-dependent autophagy but this process was mTOR independent [33]. Indeed, Ca^2+^ released to the cytoplasm through IP3-R is reported to play role in both inhibition and activation of autophagy, probably dependently on cellular state [32]. In autophagy induced by ATP, P2 purinoreceptors were stimulated to generate IP3 that triggers the release of Ca^2+^ from the ER through IP3-R [25]. Similarly, rapamycin treatment in HeLa cells increased ER Ca^2+^ store content and resulted in potentiated release through the IP3-R [32]. Furthermore, intracellular Ca^2+^ signal was essential for mTOR-dependent autophagy. It is possible though both IP3-R inhibition and activation can evoke autophagy via distinct signaling pathways. While IP3-R Ca^2+^ signaling-dependent autophagy leads to mTORC1 inhibition, it should be studied whether the blockage of IP3-R induces ER stress and UPR or if it promotes autophagy independently of these mechanisms [25].

Inhibition of voltage-dependent calcium channel T or L is also reported to evoke autophagy in cancer cells. Long-lasting, high voltage-activated (L-type) calcium channels and transient, low voltage-activated (T-type) calcium channels are often overexpressed in various cancers [34–36]. Verapamil, the L-type calcium channel blocker, induced autophagy-like process in human adenocarcinoma COLO 205 cells as judged by ultrastructural studies [37]. The other L-type calcium channel blocker, nifedipine, decreased proliferation and migration and evoked autophagy in endometrial carcinoma HEC-1A cells. Moreover, suppression of autophagy by 3-MA led to apoptosis induction [21]. As calcium inward currents regulators, T-type calcium channels play also role in cells proliferation and differentiation processes. Zhang et al. [38] described retarded proliferation and migration upon endostatin in human glioblastoma U87 cells. The mechanism of action in endostatin-treated cells occurred via blockage of T-type, but not L-type calcium currents. Furthermore, T-type calcium channel blocker, [3-(1,1′-biphenyl-4-yl)-2-(1-methyl-5-dimethylaminopentylamino)-3,4-dihydroquinazolin-4-yl]-N-benzylacetamide 2 hydrochloride (KYS 05090), induced autophagy and apoptosis-mediated cell death in human lung adenocarcinoma A549 cell line. These processes were inhibited either by bafilomycin A, a potent autophagy inhibitor, or by inhibitor of caspase 3, zVAD-fmk [35]. Regardless of decreasing intracellular Ca^2+^ levels, KYS 05090 inhibited also glucose uptake and elevated ROS generation [35].

Voltage-gated calcium channels inhibitors are known for blockage of calcium entry inside the cell; however some of them are reported to modulate autophagy in noninvolving calcium channels pathway. For instance, tetrandrine is reported to be a lysosomal deacidification agent, able to block autophagy flux at the step of lysosomal degradation. Furthermore tetrandrine induced apoptosis in PC3 cells and tumor xenografts, due to reduced glucose uptake [39]. Also verapamil and its derivatives were reported to evoke cytotoxicity on various cancer cells via distinct mechanisms including reversing multidrug resistance and inhibition of glucose import [40–42].

CRAC is a major channel contributing to changes in intracellular Ca^2+^ concentration. It consists of structural proteins from ORAI family (ORAI 1, ORAI 2, and ORAI 3) and Ca^2+^ sensor stromal interaction molecule (STIM). CRAC activation resulting in SOCE plays critical role in physiological processes of immune system cells such as T lymphocytes [43, 44]. CRAC is also important for cancer cells maintenance, usage of CRAC blockers, as well as ORAI 1 silencing, markedly inhibited cell proliferation of glioblastoma cell lines C6 (rat) and U251 (human) [45]. In HeLa cells targeting the ORAI 1 and calcium-transporting ATPase type 2C member 1 (ATP2C1) by the microRNA miR-519 resulted in their downregulation and subsequent intracellular Ca^2+^ elevation. Furthermore p21 level was increased by activation of CaMKK*β* and GSK3*β*. Altogether this resulted in cell growth arrest, autophagic phenotype, and increased cell survival [29].

## 5. Glucose Metabolism during Autophagy

Most malignant cells use glycolysis as a major pathway for ATP generation and since this process is inefficient, cancer cells exhibit abnormally high glycolytic rates to maintain ATP homeostasis [46]. Glycolysis is triggered by mutations in several oncogenes like Ras, Akt, and Myc [9, 17]. This feature of cancer cells and other immortalized cell lines is called “Warburg effect” and is mainly manifested by high glucose uptake followed by intensive lactic acid formation in the cytosol. High lactate production is desired as acidic environment favors the tumor growth [47]. In fact, tumor cells which are highly dependent on glycolysis are more sensitive to basic nutrient deprivation such as glucose [48]. In 4T1 cancer cells, glucose deprivation and blockage of lactic acidosis caused cell death within 24 hours [46]. Limited glucose access may also lead to autophagy induction. In poor nutrient conditions the mTORC1 is suppressed by AMPK and autophagy provides cells with new energy sources. In PC3 and LNCaP prostate cancer cell lines therapeutic starvation with 2DG evoked autophagy judged by presence of autophagic hallmarks such as higher expression of Beclin 1 and LC3 II, together with its membrane translocation [49]. Another example is autophagy induced by diindolylmethane (DIM) in ovarian cancer cells, mediated by ER stress and AMPK activation [50]. Activation of AMPK in pancreatic adenocarcinoma PANC-1 cell line after cannabinoids treatment was mediated by ROS elevation which led to inhibition of Akt/c-Myc pathway and consequently Krebs cycle and glycolysis downregulation. Disturbance in PANC-1 metabolism caused strong induction of autophagy and limitation in cell growth [48].

Transport of glucose across plasma membrane is a first rate-limiting step for glucose metabolism and is mediated by dedicated protein group named as GLUTs [51]. Upregulation of specific glucose transporters may play a key role in exaggerated glucose uptake in malignant cells, which is required to maintain high rate of glycolysis. GLUT 1, which is involved in glucose uptake in the basic and stress state, is expressed at elevated levels in almost all human cancers including brain, breast, head and neck, bladder, renal, colorectal, lung, and ovarian cancers [51]. Krzeslak et al. [52] reported the involvement of GLUT 1 and GLUT 3 in glucose transport in endometrial and breast carcinoma cells. Lately, GLUT 12, physiologically expressed in the insulin-sensitive tissues such as skeletal muscle, heart, and fat, has been localized in human breast and prostate cancer both intracellularly and at the plasma membrane. Moreover, this can be an example of reactivation of a gene, expressed in the embryo and downregulated in most adult tissues in nonpathological conditions [51]. Besides the GLUT family, other proteins like SGLT1 or IGFs and their receptors (IGF-Rs) are engaged in glucose transport and are often upregulated in cancer cells [53, 54]. SGLT-1 was overexpressed in colorectal cancer tissue together with EGFR and these were correlated with higher clinical stages of tumor disease [53]. IGF-I/IGF-IR is a protein that promotes cell survival, proliferation, and differentiation, whose overexpression is associated with many types of cancer including lung cancer, neuroblastoma, cervical, breast, and colon cancer [54, 55]. Moreover, IGF-I was shown to significantly increase the initial rate of glucose uptake by HT29-D4 colon cancer cells [56]. On the contrary, in Caco-2 intestinal adenocarcinoma cell line, transport of glucose occurred via an Na^+^/glucose transporter, independently of insulin and IGF-1 receptors [57]. Restriction of glucose uptake may affect the metabolism of malignant cells and limit significantly tumor growth by inducing cell death. In fact, downregulation of IGF-I/IGF-IR signaling can inhibit tumorigenesis, reverse the transformed phenotype, and induce apoptosis [55]. Next, in MM.1S myeloma cell line the decrease in glucose consumption stimulated by purine analog 8-aminoadenosine application exerted energetic stress and activation of autophagy, which played role in cell redundancy to the treatment. Cotreatment by 8-aminoadenosine and autophagy inhibitors stimulated apoptosis induction, although the effect was reversible by pretreatment with metformin and overexpression of GLUT1 [58].

Glucose transporters might be influenced by distinct factors during tumor progression. Tolerance of tumor cells to nutrient deprivation depends on deregulation of both oncogenes and oncosuppressors. Cav1 is a growth suppressor protein, although its level is often elevated in advanced cancer, suggesting the oncogenic switch to the role in growth progression. In various human colorectal tissues and cancer cell lines, inhibition of Cav1 triggered AMPK-mediated autophagy with p53-dependent G1 cell-cycle arrest. Moreover, glucose uptake, lactate accumulation, and ATP levels were reduced. Thus, overexpression of Cav1 in tested human colon cancers is thought to be responsible for their upregulated metabolism via stimulation of* Glut 3* gene expression [59, 60]. Another oncosuppressor involved in glucose metabolism regulation is HIPK2, whose activation upon several cellular stresses causes cell death [61]. In human RKO colon cancer cells harboring wt-HIPK2 (HIPK2^+^/^+^), cell death was induced, mainly due to c-Jun NH2-terminal kinase (JNK) activation upon glucose starvation. In contrast the same conditions did not induce cell death in siHIPK2, which exhibited upregulated glycolytic activity and autophagy. Although targeting glycolysis by 2-DG or siGlut-1 does not induce siHIPK2 cell death under glucose starvation, this was achieved by zinc supplementation that reversed p53 misfolding and inhibited HIF-1 activity. The cytotoxic effect in siHIPK2 RKO cells was potentiated by inhibiting autophagy, which played role in establishing tumor survival under glucose deprivation [61].

Also ER stress can affect glucose metabolism in cancer cells. In IL-3-dependent Bak^−/−^/Bax^−/−^ hematopoietic cells resistant to apoptosis, exposure to tunicamycin resulted in decreased cell surface GLUT 1 level and impaired Akt signaling, which was probably a reason for observed reduced glucose uptake and lactate production and fall in mitochondrial potential and ATP level disturbance. In the absence of apoptosis, tunicamycin evoked autophagy, which might be important for cell survival [62].

## 6. ER Stress

Endoplasmic reticulum (ER) facilitates the proper folding of the synthesized proteins and serves as a Ca^2+^ store inside the cell. ER stress appears in response to different physiological and pathological conditions, for example, when the aggregation of prone proteins occurs, glucose starvation causes limited protein glycosylation, or during hypoxia when the formation of disulfide bonds is reduced [25]. In such situations a specific nuclear signaling pathway called UPR is activated and results in reduced global protein synthesis and increased production of proteins required for proper folding at ER such as chaperones. Meister et al. [63] reported enhanced ER stress followed by cell death in myeloma cells after combined treatment with verapamil and proteasome inhibitor, bortezomib. When ER stress is being prolonged and misfolded/unfolded proteins exceed the capacity of the proteasome degradation system, it might trigger autophagy [25]. In mammalian cells UPR can be mediated by activation of different stress transducers like PERK, ATF6, or IRE1, which sense the level of unfolded proteins in ER lumen and pass the signal to cytoplasm and nucleus. Activation of PERK leads to phosphorylation of the *α* subunit of the eukaryotic initiation factor (elF2*α*), which inhibits the assembly of the 80S ribosome and inhibits protein synthesis while autophagy is induced [64]. Activation of IRE1 and ATF6 promotes the transcription of UPR target genes [65]. ER stress leads also to Ca^2+^ releasing from the ER to the cytosol, which results in activation of numerous kinases and proteases involved in autophagy, such as CaMKK*β* or DAPK. Both of them stimulate disruption of Beclin 1 inhibitory complexes (Beclin 1-IP3-R or Beclin 1-Bcl-2). Furthermore, CaMKK*β* is also an upstream activator of AMPK, which inhibits mTORC1 [6, 66]. Beclin 1 can be activated also through IRE1-JNK pathway [67].

In cancer cells, levels of misfolded proteins and ER stress are often increased because of gene mutations and stressful microenvironment. Furthermore, ER stress is frequently a cellular response to anticancer treatment. Salazar et al. [68] reported autophagy and cell death induction upon Δ^9^-tetrahydrocannabinol (THC) treatment in human glioma cells and tumor xenografts. Moreover inhibition of autophagy prevented cell death; also, autophagy deficient tumors were resistant to THC growth-inhibiting action. THC caused ceramide accumulation and elF2*α* phosphorylation leading to ER stress induction. Consequently autophagy occurred through tribbles homolog 3- (TRB3-) dependent inhibition of the Akt-mTORC1 pathway [68].

It is known that ER stress-induced autophagy depends strongly on Ca^2+^ homeostasis. Studies conducted with thapsigargin or tunicamycin, inhibitors of ER Ca^2+^-ATPase, revealed that in IRE1-deficient MEFs or MEFs treated with JNK inhibitor, autophagy induced by ER stress, was inhibited, indicating that the IRE1-JNK pathway is required for autophagy activation [65]. In HCT 116 colon cancer cells inhibition of proteasome activity by MG132 resulted in increased cytoplasmic Ca^2+^ levels, ER stress, and autophagy induction. BAPTA-AM treatment overcame Ca^2+^ elevation, ER stress, and cellular vacuolization but did not prevent from MG132-induced apoptosis [69]. These results indicate that changes in calcium homeostasis often trigger ER stress leading to UPR mediated autophagy.

It seems that distinct cellular pathways inducing autophagy might be integrated in some points. For instance, starvation-induced autophagy may also depend on IP3-R-mediated Ca^2+^ signaling [32]. ER stress-mediated autophagic pathways also integrate various cellular stresses. Ca^2+^ perturbations as well as glucose starvation may lead to similar cellular response as ER stress and UPR activation.

During maturation the majority of proteins require Ca^2+^ to proper folding. It is also known that 80% of proteins synthesized in rough ER undergo glycosylation [70] and highly depend on glucose availability. Treatment with glucose analog, 2-DG, is known to induce ER stress-mediated autophagy in pancreatic, melanoma, and breast cancer cell lines. Further, 2-DG not only blocked glycolysis, thereby lowering levels of ATP, but also impaired glycosylation, as addition of exogenous mannose was able to reverse ER stress and autophagy [71]. In turn, in HT-29 human colonic carcinoma cells, high rate of N-glycosylated proteins substituted with ER glycans is degraded in autophagy pathway. Inhibition of ER glucosidases stabilized freshly synthesized N-linked glycoproteins and inhibited their degradation via macroautophagy. In this case autophagy seems to be a selective process connected with ER-associated quality control of synthesized N-glycoproteins [72].

## 7. ER Molecular Chaperones and Transcription Factors in Autophagy Regulation

N-Glycosylation of newly synthesized proteins takes place in ER lumen and ER-associated molecular chaperones are involved in the quality control of this process. Firstly, during folding, polypeptides are recognized by ER chaperones such as 78 kDa glucose-regulated protein GRP78/BiP, calnexin/calreticulin [72], and proteins involved in the disulphide bond formation like PDI [73]. Interaction with molecular chaperones lasts till they are exported to Golgi apparatus to undergo further proceedings. The misfolded/unfolded proteins are directed to ER-associated degradation (ERAD) system where they are degraded via ER-attached proteasomes [72]. ATF4, ATF6, and XBP-1 (transcription factor targeted by IRE 1) upregulate ER chaperones, folding enzymes, and protein degradation molecules, which either prevent the aggregation of unfolded proteins or assist in their degradation [74]. In HT-29 human colon cancer cells ER stress was induced by compound K. This process was mediated via increased expression of ER chaperone GRP78/BiP and proapoptotic protein-CHOP, possibly as a consequence of PERK and IRE 1 phosphorylation and ATF 6 cleavage to active form [75].

The main UPR-upregulated protein is GRP78/BiP. It is involved not only in proteins proper folding but also in transport of proteins across ER membrane, regulation of proliferation, tumor progression, angiogenesis, autophagy, chemosensitivity, and apoptosis [73, 76, 77]. Levels of GRP78/BiP mRNA and protein are modulated by glucose availability and Ca^2+^ concentration [78–80]. Under physiological conditions GRP78/BiP is attached to ATF6, IRE1, and PERK protein, residing in ER membrane. When quantity of misfolded/unfolded proteins exceeds the ER lumen capacity, GRP78/BiP dissociates from its binding partners so they have ability of autophosphorylation and activate the UPR reaction [81]. Elevated level of GRP78/BiP was observed in human nasopharyngeal carcinoma cells during ER stress-mediated autophagy induced after DDP, 2-DG, ionizing irradiation, and tunicamycin treatment. Autophagy was activated as a protective mechanism; therefore, using 3-MA contributed to apoptosis induction [82]. GRP78/BiP is also a key player in autophagy induced in tumors from BRAFV^600E^ melanoma patients treated either with B-Raf inhibitor or with combined B-Raf and MEK inhibition. Furthermore, autophagy level was significantly higher in B-Raf inhibition-resistant tumors. Induced autophagy is mediated via mutated B-Raf bounding to GRP78/BiP allowing the subsequent PERK phosphorylation. This data provides the possible mechanism of B-Raf mutation-driven myeloma tumors resistance to B-Raf inhibition therapy [83]. Moreover Li et al. [74] showed that GRP78/BiP is required for UPR activation and following autophagy in HeLa cancer cells. Inhibition of GRP78/BiP by siRNA resulted in blockage of autophagosome formation upon ER stress or nutrition starvation. Impaired autophagy was recovered after simultaneous knockdown of GRP78 and XBP-1, which are known to regulate ER functions [74]. However, Bennett et al. [81] reported androgen receptor-mediated temporary upregulation of GRP78/BiP in prostate cancer LNCaP cell line upon chronic serum starvation, which contributed to ER stability and the delay in onset of autophagy and cell death execution.

Other ER stress-upregulated protein is CHOP, a transcription factor implicated in the control of translation and apoptosis, downstream target of PERK and ATF4 [74]. Induced by UPR, CHOP protein may also contribute to autophagy induction by downregulating the Bcl-2 expression [25, 73]. It is also known that ATF4 and CHOP activate expression of two genes essential for autophagy: ATF4 binds to the promoter of* Map1lc3B*, while CHOP activates the transcription of* Atg5 *[84]. 

Hsp27 is stress-activated multifunctional chaperone that inhibits treatment-induced apoptosis and causes therapy resistance. It is expressed mainly in cytoplasm but occurs also in ER and nucleus. Hsp27 is present in many cancer types, for example, in castration-resistant prostate cancer (CRPC). Using OGX-427, a second-generation antisense inhibitor of Hsp27, Kumano et al. [85] confirmed that Hsp27 reduced proteasome inhibitor-induced ER stress and accumulation of misfolded/ubiquitinated protein levels, mainly by increasing proteasome activity and/or stabilization client-protein complexes. However, inhibition of Hsp27 led to suppression of ubiquitin-proteasome system and activation of ER stress and UPR. Moreover, Hsp27 knockdown induced cytoprotective autophagy, yet combined inhibition of Hsp27 and autophagy further disrupted proteostasis and caused apoptosis in prostate cancer cells [85].

CLU is a heterodimeric, highly conserved, disulfide-bond glycoprotein. During maturation secretory form of CLU (sCLU) undergoes heavy glycosylation which contributes to its cytoprotective role and possibly protects from its aggregation [86], whereas nonglycosylated nuclear CLU (nCLU) plays role in apoptosis induction [87]. Because CLU maturation is complex and highly depends on processing in ER lumen and in Golgi apparatus, CLU seems to be extremely sensitive to ER stress. Lately, Kang et al. [88] reported other nonglycosylated variants of CLU accumulated in ER, confirming the role of N-glycosylation in preventing from terminal misfolding of CLU protein. Moreover N-glycan deficient CLU induced cytotoxicity may be a reason for disease pathogeneses associated with chronic ER stress [88]. CLU function is also often associated with intracellular Ca^2+^ homeostasis. It is assumed that in Ca^2+^ deficiency CLU translocates to nucleus to intermediate apoptosis induction, while in elevated levels of Ca^2+^ in cytosol it is mainly secreted to play cytoprotective role [89]. Little is known about CLU role in autophagy, although its involvement in carcinogenesis, tumor survival, regulation of adhesion, cell cycle, and apoptosis has been widely described [87, 90, 91]. Increased levels of CLU have been reported in several malignancies, including breast, colon, lung, and prostate cancers [92, 93]. CLU function as an extracellular and intracellular chaperone during ER stress was also reported. Wyatt et al. [94] described CLU to form stable complexes with misfolded client proteins and target them to lysosomal degradation both* in vitro* and* in vivo*. Balantinou et al. [95] confirmed that CLU is degraded by both proteasome and lysosome systems. In TRAMP mouse model of prostate cancer, phenethyl isothiocyanate treatment inhibited carcinogenesis through autophagy induction and decreased level of CLU protein. CLU was then identified as a potential plasma biomarker of phenethyl isothiocyanate-induced chemopreventive activity [92].

## 8. Conclusions and Current Perspectives in Anticancer Therapy

Primary function of autophagy process is the maintenance of cellular energetic status while coping with numerous affecting stresses. During tumor development autophagy is often used to clear defective proteins and organelles. Moreover, tumor cells take an advantage of autophagy and maintain cell survival in response to anticancer treatments [8]. Numerous studies were undertaken to evoke molecular mechanisms leading to autophagy induction in cancer cells as well as its downstream consequences including contribution to various cell death processes. It is known that autophagy might be modulated by changes in ion homeostasis and metabolic perturbations, which may alter intracellular biochemical reactions and proceed in further induction of cellular stress signaling pathways. Ca^2+^ ion channels and glucose transporters can mediate signal transduction from extracellular to intracellular space and therefore participate in ER stress induction, which is one of possible ways to evoke autophagy. This opens the possibility of using Ca^2+^ ion channels and glucose transporters modulators in control of autophagy for therapeutic purposes. In mice with acute pancreatitis the utilization of 2APB, an antagonist of IP3-R, extenuated pathological changes, possibly via blockage of autophagy [96]. In turn, glucose analog, 2-DG, responsible for autophagy induction upon inhibition of glycolysis and insufficient ATP levels, is reported to evoke cell death when combined with antimycin A or metformin in various cancer cell types. Moreover combination of 2-DG and docetaxel or radiotherapy is currently on trials for therapy for glioblastoma, lung, and breast cancers [97]. Research is being undertaken on establishing new therapy based on autophagy activation via, for example, ER stress induction (sorafenib), mTOR inhibition (aurora kinase A), or AMPK activation (atorvastatin) in various cancer types [28]. Zhao et al. [98] showed in preclinical studies on nasopharyngeal carcinoma (NPC) model, that upon AKT inhibition by MK-2206, autophagy, but not apoptosis, was induced in CNE-2 cell line. Moreover MK-2206 inhibited the growth of tumor while CNE-2 cells were implanted into nude mice.

Modulation of autophagy seems to be promising in anticancer therapy; however, it should be further investigated whether therapeutic induction of autophagy might potentiate chemoresistance in tumor cells or contributes to cell death. The role of autophagy in cancer is complex and especially depends on cancer type and stage of development. In a mouse model of non-small-cell lung cancer (NSCLC)* Atg7* deletion did not affect tumor formation caused by kRas activation and p53 deletion. However, in mice with established tumors, deletion of* Atg7* blocked tumor progression and led to tumor cell death, before normal tissue destruction [99]. These findings indicate that depletion of autophagy might be a useful tool in providing the time frame for destructing tumor tissue. Autophagy impairment might also play a beneficial role in response to ionizing radiation (IR). Deletion of* Atg5* sensitized human and mouse cancer cell lines to induced cell death and inhibited* in vivo* tumor growth in immunodeficient mice. However, in immunocompetent mice, autophagy depletion decreased therapeutic effect of IR [100], indicating a context-dependent matter in autophagy modulation. Recently Rosenfeldt et al. [101]. showed in a humanized genetically modified mouse model of pancreatic ductal adenocarcinoma (PDAC) that autophagy role in tumor development depends on the status of the tumor suppressor p53. In mice bearing oncogenic kRas, deletion of* Atg5* and* Atg7 *inhibited tumor development. On the contrary, in mice containing oncogenic kRas but lacking p53, deletion of autophagy genes accelerated tumor formation. It is also proved that in this case blocking of autophagy with hydroxychloroquine contributed to tumor progression. These results are important in view of prospective anticancer treatment targeting autophagy. In particular, many current clinical trials cover therapies combining chloroquine or hydroxychloroquine with classical chemotherapy agents such as DDP or bortezomib [28]. Chloroquine was also shown to induce cell death in mice glioblastoma model in combined treatment with mTOR inhibitor, CC214-2 [102].

Autophagy ability to perform detoxification seems to occur frequently after anticancer drugs application. Therefore autophagy modulation might be a prospective tool in anticancer therapy, although further studies are of a high importance to uncover the circumstances when autophagy inhibition or activation may be implemented into clinic.

## Abbreviations

2DG: 2-Deoxy-D-glucose
3-MA: 3-Methyladenine
5-FU: 5-Fluorouracil
Ambra 1: Activating molecule in Beclin 1 regulated autophagy
AMPK: AMP-activated protein kinase
ATF 4: Activating transcription factor 4
ATF 6: Activating transcription factor 6
*Atg*: Autophagy-related gene
ATP2C1: Calcium-transporting ATPase type 2C member 1
Bad: Bcl-2 associated death
Bax: Bcl-2-associated X protein
Bcl-2: B-cell lymphoma 2
Bcl-X_L_: B-cell lymphoma extra large
Bif1: B1/BAX-interacting factor 1
Bif-1: Bax interacting factor 1
CaMKK*β*: Ca^2+^/calmodulin-dependent kinase *β*
Cav1: Caveolin 1
cFLIP: Cellular FLICE-like inhibitory protein
CHOP: C/EBP-homologous protein
CLU: Clusterin
CRAC: Ca^2+^ release-activated Ca^2+^ channel
DAPK: Death-associated protein kinase
DDP: Cis-diamminedichloroplatinum (II)
DRAM: Damage regulated autophagy modulator
EGFR: Epidermal growth factor receptor
elF2*α*: Eukaryotic initiation factor
ER: Endoplasmic reticulum
ERK1/2: Extracellular signal-regulated kinases 1/2
FIP200: Focal adhesion kinase (FAK) family interacting protein of 200 kDa
GLUT: Glucose transporter
HIF-1: Hypoxia-inducible factor 1
HIPK2: Homeodomain-interacting protein kinase 2
Hsp27: Heat shock protein 27
IGFs: Insulin-like growth factors
IP3-R: Inositol triphosphate receptor
IRE1: Inositol-requiring enzyme 1
JNK: c-Jun NH2-terminal kinase
MAP LC3: Microtubule-associated protein 1 light chain 3
MEK1/2: Mitogen-activated protein kinase 1/2
mTOR: Mammalian target of rapamycin kinase
mTORC1: Mammalian target of rapamycin complex 1
PARP-1: Poly[ADP-ribose]polymerase-1
PDI: Protein disulfide isomerase
PERK: RNA-dependent protein ER kinase
PI3-K: Phosphoinositide 3-kinase
PMCA: Plasma membrane calcium ATPase
PUMA: p53 upregulated modulator of apoptosis
Raf-1: Ras protooncogene serine/threonine protein kinase
ROS: Reactive oxygen species
RyR: Ryanodine receptor
SERCA: Sarco-/endoplasmic reticular calcium ATPase
SGLT: Na^+^-dependent glucose cotransporter
SOCE: Store-operated calcium channel entry
TFEB: Transcription factor EB
ULK 1/2: Unc-51-like kinase 1/2
UPR: Unfolded protein response
UVRAG: Ultraviolet irradiation resistance-associated gene product
XBP-1: X-box binding protein 1.
